# Psychological Well-Being During Adolescence: Stability and Association With Romantic Relationships

**DOI:** 10.3389/fpsyg.2019.01772

**Published:** 2019-08-02

**Authors:** Mercedes Gómez-López, Carmen Viejo, Rosario Ortega-Ruiz

**Affiliations:** Department of Psychology, Universidad de Córdoba, Córdoba, Spain

**Keywords:** self-acceptance, positive interpersonal relationships, autonomy, life development, longitudinal study, structural equation modeling

## Abstract

The concept of well-being is complex and is in common use not only in the area of health but also in the field of human development. Well-being depends on both the individual and the environment, and during childhood and adolescence, the environmental factor can be decisive. Family, school, and peers are widely recognized as significant contexts for successful development, but romantic context is also undoubtedly important. Romantic relationships constitute a new dimension in the adolescent’s social life, but little attention so far has been paid to their importance in well-being. Defined as developmental tasks, they have been associated both positive and negative outcomes, although their impact on well-being has not yet been clarified. This study uses a eudaimonic approach to evaluate four dimensions of psychological well-being: self-acceptance, positive interpersonal relationships, autonomy, and life development, and has a two-fold objective: (1) to analyze adolescents’ levels of psychological well-being and their stability over time, and (2) to analyze the association between romantic relationships and adolescents’ psychological well-being. Using a longitudinal design, we analyzed data from a sample of 747 adolescents from Andalusia (Spain) between 13 and 17 years old (50.5% girls, mean age wave 1 = 14.55, SD = 0.84). The results revealed medium to high levels of psychological well-being, which remained stable throughout the time of the study, and an increase in wave 2 only in positive interpersonal relationships dimension. The Structural Equation Modeling analysis showed romantic relationships as a predictor of psychological well-being, having a positive link with positive interpersonal relationships and with life development, and a negative link with autonomy and self-acceptance. These results are discussed in terms of the need to use approaches focused on the specific characteristics and evolution of well-being during adolescence, as well as on the importance of considering romantic relationships as developmental assets which have the potential to influence well-being during these years.

## Introduction

Psychology has traditionally conceptualized individuals in terms of psychopathology, dysfunction, and failure (Seligman and Csikszentmihalyi, 2000). It has been focused on repairing damage following a disease model of human functioning, paying little attention to the building of positive qualities (Seligman and Csikszentmihalyi, 2000). In this sense, adolescence has not been an exception. Since it was established as an empirical field of study at the beginning of the twentieth century, it has been normally viewed as a period of life beset with problems and difficulties (Steinberg and Morris, 2001), collecting research large amount of data related to risk factors, problem behavior, and prevention formulas (Oliva et al., 2010; Dvorsky et al., 2018). As a result, knowledge about aspects related to optimal functioning and positive development during these years is significantly smaller (Huebner et al., 2009), contributing to the conception that a healthy adolescence is one without problems (Oliva et al., 2010). However, different perspectives have emerged and advocate understanding it from a broader and more balanced perspective, considering that optimal functioning during adolescence is more than the absence of problems, difficulties or pathologies (Lerner et al., 2005; Seligman, 2008). These include positive psychology, which considers the potential of adolescents to achieve a healthy and successful development, adopting a well-being-oriented approach (Seligman et al., 2009).

## Conceptualization of Well-Being

The emergence and growth of positive psychology has led to an increase in well-being research, which has produced two theoretical approaches: hedonic and eudaimonic (Ryan and Deci, 2001). The hedonic view reflects the notion of well-being as an outcome, consisting of an internal state of pleasure or happiness, and focuses on subjective well-being (Ryan and Deci, 2001; Pavot and Diener, 2008; Ryan et al., 2008). From this perspective, well-being is defined in terms of experiencing high levels of positive affect, low levels of negative affect, and a high degree of satisfaction with life (Deci and Ryan, 2008). The eudaimonic view, on the other side, posits that well-being consists of more than just happiness. Eudaimonic theories consider that not all desires – outcomes that a person might value – would lead to well-being when they are achieved (Ryan and Deci, 2001). From this point of view, well-being is not an outcome or final state, but a process of fulfilling human potentials (Deci and Ryan, 2008). It concerns to positive functioning and to the development of capacities and virtues (Ryan and Deci, 2001; Ryan et al., 2008). In this field, Ryff’s multidimensional model of psychological well-being has received the most empirical support (Ryff, 2014). According to this approach, well-being is made up of six dimensions: (1) *autonomy*, or the ability to regulate our own behavior, resist social pressure, and follow our convictions, even if they go against the general opinion; (2) *environmental mastery*, or the ability to manage the context and daily activities; (3) *personal growth,* which includes a continuous process of developing our own potential, the ability to be open to new experiences and the feeling of improving over time; (4) *positive relationships with others*, defined as the establishment of close, trusting and meaningful bonds with others, as well as showing concern for the well-being of others, and the expression of empathy, affection, and intimacy; (5) *purpose in life*, or setting objectives and goals which give meaning and direction to our lives; and (6) *self-acceptance*, or the ability to have a positive attitude and feelings of satisfaction and acceptance of ourselves, including both our good and bad qualities. Each of these dimensions represents what it means to be healthy, well and fully functioning, and articulates the different challenges that people face in their effort to achieve positive functioning (Ryff and Singer, 2008). In other words, people try to view themselves positively although they are aware of their own limitations (self-acceptance), seek to maintain satisfactory interpersonal relationships (positive relations with others), develop a sense of self-determination and personal authority in their interaction with the context (autonomy), make the most of their own talents and abilities to achieve their goals (personal growth), manage their environment to meet their needs (environmental mastery), and find meaning in the effort they make and the challenges they encounter in life (purpose in life) (Keyes et al., 2002).

According to previous studies analyzing the effects of hedonic and eudaimonic activities on well-being, eudaimonic seem to produce more long-term effects than hedonic (Steger et al., 2008; Huta and Ryan, 2010). Research suggests that hedonically motivated activity makes people feel good at the immediate or short-term time scale; that is, it may increase levels of pleasure or satisfaction perceived while it is being carried out, but it may not lead to positive affect or high life satisfaction in the long run (Huta and Ryan, 2010). On the contrary, eudaimonic activity may have more cumulative and enduring effects on well-being (Steger et al., 2008; Huta and Ryan, 2010). For example, cultivating trusting and healthy relationships can create a resource that increases people’s quality of life (Gable et al., 2004). As people build resources such as feelings of mastery, competence or better relationships, they are more likely to perceive their life as satisfying and meaningful (Steger et al., 2008). In other words, eudaimonic activity is especially likely to build durable resources, leading to more fulfilled people with stable levels of well-being (Steger et al., 2008).

## The Study of Adolescent Well-Being

Previous research, mainly focused on the adult population, has shown that psychological well-being is a reliable predictor of health and long-term positive adjustment (Ryff, 2017). People with higher levels of well-being suffer fewer illnesses, have an increased life expectancy and engage in more healthy behavior (for a review, see Ryff, 2017). However, longitudinal studies have also shown that dimensions such as personal growth and purpose in life tend to decline as we get older (Clarke et al., 2000; Springer et al., 2011). Although research focused on adolescence is limited, a number of longitudinal studies have been found providing evidence about the evolution of well-being in this period. The World Health Organization (WHO), through the *Health Behaviour in School-aged Children* (HBSC) study, collects data every 4 years about children aged 11, 13, and 15 in over 40 North American and European countries, understanding well-being in terms of satisfaction with life (Inchley et al., 2016). As regards gender, the results of this research showed that well-being in adolescents decreased with age in both sexes, and that boys generally showed better levels of satisfaction with life than girls (Inchley et al., 2016). One of the conclusions of HBSC study stresses the importance of interpersonal relationships in adolescent well-being and states that peers and parents play a key role as protective assets in young people’s lives. Along the same lines, the research carried out by Patalay and Fitzsimons (2018) in a sample of 9,553 adolescents aged 11 and 14, found well-being to be highly unstable, and suggested that girls were more likely to experience a decline in well-being over time. Well-being was measured in terms of satisfaction with different domains of life, including school, family, friends, schoolwork, appearance, and life as a whole (Patalay and Fitzsimons, 2018). Important predictors of these results were lower family income, a poorer relationship with parents, lower school connectedness, and greater cognitive ability. Booker et al. (2018) also found a decline in well-being over time, especially among girls. To measure well-being, authors used happiness with six domains of life (i.e., friends, family, appearance, school, schoolwork, and life as a whole) as a key variable and included a measure of emotional and behavioral problems (Booker et al., 2018). According to these authors, girls as they grow up tended increasingly to compare themselves socially with others and perceive that the others are better than them, which could lead to lower levels of well-being compared to boys (Booker et al., 2018). In contrast, Lerkkanen et al. (2018), examining patterns and dynamics of pattern change of 1,666 students’ psychological well-being, found that the different profiles of well-being (e.g., high, average, low well-being profile) tended to improve over time, and that future expectations or aspirations were a critical variable. Although this study claims to analyze psychological well-being, it is necessary to clarify that it does not follow a eudaimonic approach. Well-being was operationalized to consist of school enjoyment, future educational aspirations, (absence of) school burnout, self-esteem, and (absence of) externalizing and internalizing problems. Meade and Dowswell (2016) also reported that the general well-being of adolescents, in terms of Health-Related Quality of life (HRQoL), usually remained relatively stable over time, although, again, gender differences were found, with worse predictions for girls. According to the authors, the change in personal standards used to self-assess quality of life accounts for these differences. The HRQoL construct represented the overall well-being of the participants, measured through five dimensions: physical well-being, psychological well-being, autonomy and parents’ relations, social support and peers, and school environment. Focusing on psychological well-being, it was assessed using hedonic elements, such as positive emotions, satisfaction with life, and the absence of loneliness and sadness. Finally, from a sociodemographic point of view, Ottová-Jordan et al. (2015), analyzing data from HBSC study, found different tendencies depending on the country. For example, in countries such as Spain, Croatia or Greece, the cohorts showed a steady decrease in well-being (satisfaction with life); in Denmark, Finland or Norway, there was a linear increase, while in Austria, Canada or Scotland, there was a clear U-shaped trend (Ottová-Jordan et al., 2015). The authors attribute these results to circumstances such as concerns about the future, high expectations set by their context (family, school, and peers), school pressure, or country-specific characteristics (e.g., economic situation, unemployment rates, social insecurity), which can result in higher levels of perceived stress.

Despite this evidence, it can be observed that these studies mainly adopt a hedonic perspective. As far as we know, there are only a few cross-sectional studies which adopt a eudaimonic approach to the study of adolescent well-being. Fernandes et al. (2010) developed a validation of the Ryff’s scales for Portuguese adolescents, achieving a new version with good adjustment indices. The age of the participants was between the ages of 10 and 18 years old, and their well-being levels were medium-high in all the dimensions in study 2; however, lower scores were obtained in study 1. In another work (Fernandes et al., 2011), researchers focused on the relationship between school satisfaction and psychological well-being of 698 adolescents between 12 and 18 years old, finding a positive correlation between both variables. Psychological well-being was measured using Ryff’s scales. Well-being scores showed medium-high levels, with the highest corresponding to positive relationships with others and personal growth. Loera-Malvaez et al. (2008) also adapted Ryff’s scales for Mexican adolescents, while Opree et al. (2018) did the same for children ages 8–12, finding medium scores in all dimensions. Ruini et al. (2009) tested the efficacy of a school program for the promotion of psychological well-being in adolescent with a mean age of 14.4 years. The results showed medium scores in all dimensions prior to intervention, finding an increase in personal growth in the follow-up. Finally, Vescovelli et al. (2014) described the psychological well-being of 150 adolescents aged 13–19, and its relationship to distress and prosocial behavior. Well-being was again measured with Ryff’s scales, obtaining participants medium scores in all dimensions. No significant differences emerged according to gender and age did not show a significant effect as well.

Notwithstanding the foregoing, it has been previously established that research regarding the judgements of children and adolescent about their life satisfaction has received an increasing amount of attention (Huebner, 2004), but that psychological well-being at these stages remains an unexplored field of research (Opree et al., 2018) which has gathered less empirical effort (Ryff, 2014). For these reasons, to obtain a rigorous scientific knowledge of how well-being operates during adolescent years, empirical research must include not only perceptions about global satisfaction with life or affective balance, but also the role of the quest for meaning, self-realization and the efforts young people make to thrive, flourish, and achieve optimal development (Ryff, 2014). To this end, this study focuses on four of the six dimensions of Ryff’s psychological well-being model: self-acceptance, positive relations with others (renamed *positive interpersonal relationships*), autonomy, and personal growth (renamed *life development*) (see Viejo et al., 2018, for a review).

## The Importance of Romantic Relationships for Adolescent Well-Being

The establishment of mutually beneficial relationships between adolescents and their context is an important component for positive development and well-being during this period (Geldhof et al., 2013). Up to now, great efforts have been made to analyze how the relationships adolescents have with their school, family, neighborhood or their peers, in general, can contribute positively to their development (Benson and Faas, 2014), and it has been suggested that an optimal healthy development can be achieved if the strengths which adolescents possess can be matched by the resources existing in these contexts (Geldhof et al., 2013). However, another more specific social context has received little empirical attention: romantic relationships (Steinberg and Morris, 2001).

Romantic relationships are a key component and prevalent part of social development (Carver et al., 2003; Connolly and McIsaac, 2009). Fully embedded in the adolescents’ context (Collins and Van Dulmen, 2006) are dyadic in nature and distinctive in character. Unlike other types of relationships, such as friendships, they are commonly marked by mutual expressions of affection, a unique intensity and generally involve powerful attraction and a sexual component between their protagonists (Collins, 2003; Collins et al., 2009; Furman and Collins, 2009). Previous studies have shown that adolescents have clear conceptions of the properties that distinguish romantic relationships from friendships (Connolly et al., 2004); in other words, whereas romantic relationships are conceived in terms of passion and commitment, friendships are characterized by affiliation (Connolly et al., 1999). Defined as “on-going voluntary interactions that are mutually acknowledged, rather than identified by only one member of a pair”, the term “romantic relationships” refers to romantic status, and to the connection between two partners (Collins, 2003; Collins and Van Dulmen, 2006; Collins et al., 2009; Connolly and McIsaac, 2009).

In the change from early adolescence to late adolescence, romantic relationships play an increasingly important role (Furman, 2002) and provide an important context of support, companionship, and intimacy (Bouchey and Furman, 2003; Shulman et al., 2011), with romantic partners becoming one of the main emotional bonds (Collins and Van Dulmen, 2006; Collins et al., 2009; Connolly and McIsaac, 2009). Some studies have identified romantic relationships as contexts with a strong potential to promote positive adaptation and high levels of well-being (Collins and Van Dulmen, 2006; Furman et al., 2009; Kansky and Allen, 2018). Romantic involvement, the quality of the relationship or the positive sexually related experiences within a romantic relationship, all seem to promote lower rates of alienation and isolation, a better self-image, better future expectations, higher levels of self-esteem, and a greater level of commitment in later relationships (Ciairano et al., 2006; Viejo et al., 2015; Hensel et al., 2016). High levels of well-being are also associated with the ability to maintain beneficial interpersonal relationships, have a greater number of friends or take part in more social activities (Diener and Seligman, 2002). However, it is also necessary to admit that they can be a challenge (Larson et al., 1999; Davila, 2008), in the sense that they represent new contexts in which adolescents have no previous experience and for which they may lack the strategies needed to manage them successfully. Research suggests that the influence of romantic relationships on well-being depends largely on the level of competence and skills which these adolescents already possess (Davila et al., 2017), as well as on the way the learning process evolves; in other words, how adolescents response to a task improves each time it is repeated (Miller and Geraci, 2011).

Given this evidence, it seems certain that empirical interest in adolescent romantic relationships has increased in the last decade, as stated previously by some authors (e.g., Furman and Collins, 2009). Nevertheless, there are two main difficulties when the research goal is to understand their association to adolescent well-being: on the one hand, most of the research about this topic has been addressed in adult population (e.g., Kamp Dush and Amato, 2005; Demir, 2010). On the other hand, existing research focused on adolescence has been mainly guided by a deficit perspective, linking romantic relationships to a whole range of negative outcomes, such as emotional disorders, substance use, poor school performance or internalizing, and externalizing behaviors (e.g., Zimmer-Gembeck et al., 2001; Ackard and Neumark-Sztainer, 2002; Callahan et al., 2003; Cui et al., 2012; Viejo et al., 2015). Although there has been an increase in the amount of studies analyzing well-being during adolescence and its association with romantic relationships, it is not possible to state unequivocally that they adopt the framework provided by positive psychology regarding to the empirical approach to well-being. Studies analyzing subjective well-being seem to be aligned with positive psychology postulates, since it can be observed the use of satisfaction with life or positive and negative affect as measurement constructs (e.g., Moksnes and Haugan, 2015; Kansky et al., 2016). However, research focusing on psychological well-being has frequently used symptoms of depression and anxiety as indicators of well-being (e.g., Callahan et al., 2003; Bauermeister et al., 2010; Baams et al., 2014; Miller, 2014). These data suggest that accurate scientific knowledge about the role of romantic relationships in adolescent well-being is still sparse and reveal the need for a more balanced approach to the study of this developmental task, given its potential to influence well-being of boys and girls (Kansky and Allen, 2018). At the same time, bring to light the need to make rigorous empirical efforts in the study of well-being, especially psychological well-being, usually understood as the absence of clinical symptoms.

## The Present Study

Given the lack of empirical research on adolescent well-being, especially in relation to longitudinal data, the first aim of this work was to analyze adolescents’ level of psychological well-being and its stability over time. In addition, since there are no studies, as far as we know, that analyze the relationship between romantic relationships and well-being including an eudaimonic approach, the second aim of this study was to analyze the association between romantic relationship status and adolescents’ psychological well-being. Based on the literature presented in previous sections, two hypotheses were proposed:

Hypothesis 1: Participants will show moderate levels of psychological well-being at time 1, with no significant gender differences. At time 2, scores for all dimensions will decrease, and participants will show gender differences.

Hypothesis 2: Romantic relationships will significantly predict adolescent psychological well-being, showing a positive association with self-acceptance, positive interpersonal relationships and life development dimensions, and negative with autonomy.

## Materials and Methods

### Participants

At wave 1, the sample consisted of 747 adolescents (50.5% girls, 49.5% boys), attending both public and private schools in the Autonomous Community of Andalusia (Spain), whose age ranged from 13 to 17 years old (mean age = 14.55; SD = 0.84) (*N*_wave 2_ = 743; age range 13–18 years; mean age = 15.12; SD = 0.87).

The schools which took part were selected using random probabilistic sampling, stratified by conglomerates, single-stage and with proportional affixation (Cea D’Ancona, 2004). The strata established were the geographical area (Eastern or Western Andalusia), the school ownership (private or public), and the inhabitants (<10,000 inhabitants, 10,001–100,000 and >100,000). To measure the number of schools needed, the total sample obtained was divided by the formula proposed by Cea D’Ancona (2004), between the average number of students per group given in the statistics provided by the Andalusian Government’s Ministry of Education, choosing only one class per course and taking into account the fact that towns with under 3,000 inhabitants only had one class per academic course. The final total was 28 schools, with an assumed sampling error of 2.5% and an additional 15% to compensate for any data losses. Finally, the schools were randomly selected.

### Measurements

#### Psychological Well-Being

Psychological well-being was measured through the Brief Scale of Psychological Well-Being for Adolescents (BSPWB-A) (Viejo et al., 2018). This instrument consists of and adaptation for adolescents of the psychological well-being scales developed by Ryff (2014), validated in Spanish. The scale contains 20 items evaluated on a Likert scale of six points (1 = completely disagree to 6 = completely agree), which measures the degree of agreement with different questions relating to four dimensions: self-acceptance (e.g., *In general, I feel confident and positive about myself*) (*α*_wave 1_ = 0.85; *α*_wave 2_ = 0.89); positive interpersonal relationships (*α*_wave 1_ = 0.68; *α*_wave 2_ = 0.76); autonomy (e.g., *I often change my mind about decisions if my friends or family disagree*) (*α*_wave 1_ = 0.79; *α*_wave 2_ = 0.80); and life development (e.g., *I think everything we experience is an opportunity to grow and to become a better person*) (*α*_wave 1_ = 0.79; *α*_wave 2_ = 0.82). It is required to highlight that *positive interpersonal relationships* dimension does not perform any measure related to romantic relationships. It is focused in friendships and relationships in general (e.g., *I know that I can trust my friends, and they know that they can trust me; I do not have many people who want to listen when I need to talk*).

#### Romantic Relationship Status

Participants’ romantic relationship status was measured using one item from an adapted version of the Dating Questionnaire developed by Connolly et al. (2004). This instrument has been previously used with Spanish adolescents (Ortega et al., 2008; Sánchez et al., 2008; Viejo et al., 2015). Adolescents were asked to indicate their romantic relationship status (current romantic relationship; past romantic relationship, but not current; never had a romantic relationship) by specifying which of the responses best described their current status (i.e., *In this moment I have a partner; Currently I do not have a partner, but I have had a partner in the last 2 months; Currently I am not dating with anyone, but I have had a partner more than 2 months ago; I have never been dating with anyone*).

### Procedure

Ethical approval for the study was obtained from the Comité de Bioética y Bioseguridad de la Universidad de Córdoba (Bioethics and Biosafety Committee of the University of Cordoba) and developed in accordance with the considerations of the Declaration of Helsinki and the Spanish Society of Psychology. The study was approved by the school boards, and the consent obtained from the parents of the participants was both written and informed. Participants were visited and the anonymous, confidential and voluntary nature and the objective of the study were explained before the survey was taken. The data were collected in two waves, with the first in October 2015 and the second in May 2016. The questionnaire was conducted during school hours and filled in individually by each participant. The average time required to complete the questionnaires was approximately 20 min. For both waves, the data collection process was conducted by specialized personnel from outside the schools, who followed a strict procedure in the administration, processing, and use of the collected data.

### Data Analysis

Descriptive analysis and comparison of means were carried out using SPSS 20.0 statistical software. Regarding the first research aim, these analyses took into account the participants’ gender, and Student’s *t*-test for related samples was used, as this test is robust in cases of non-compliance with normality (Schmider et al., 2010). In relation to the second aim, comparisons of means were performed according to romantic relationship status, therefore Chi-Square test was applied.

Due to the characteristics of the questionnaires, some of them were not fully completed. Because missing data or attrition may lead to parameter bias (Nicholson et al., 2015), it is necessary to know why these data are missing. A distinction is made between three general processes that may cause missing data: missing completely at random (MCAR), missing at random (MAR), and missing not at random (MNAR) (Rubin, 1976; Little and Rubin, 1989; Little, 1992). According to Nicholson et al. (2015), Little’s MCAR test distinguishes whether missing observations are MCAR or dependent on other variables (MAR), providing a global test statistic by simultaneously testing for mean differences across all variables. This test uses a chi-squared statistic to summarize the standardized mean difference between each variable’s subgroup means and the overall mean. A significant chi-squared would suggest a significant deviation in mean differences on one or more variables between subgroups, and consequently a rejection of the null hypothesis that the data are MCAR (Nicholson et al., 2015).

In order to analyze the association between romantic relationship status and psychological well-being, the second aim was addressed by carrying out an analysis of Structural Equation Models (SEM) using the EQS 6.2 software. Here, following the recommendations of Springer and Hauser (2006), the items were identified as categorical, in order to estimate the model more accurately. Taking into account the nature of the variables and the absence of normality, the Least Squares (LS) estimation method with robust correction was used (Bryant and Satorra, 2012). In addition, the Lagrange Multiplier Test (LM Test) was performed to evaluate the statistical feasibility of specifying restrictions in the model (Byrne, 2006). One of the applications of this test is to assess the appropriateness of establishing error covariances, previously freely estimated, to identify which parameters would lead to a significantly better-fitting model (Byrne, 2006). To do that, if any of the univariate tests yield statistically significant results, LM test proceeds with a multivariate test of fixed parameters. As such, it uses a forward stepwise procedure that selects, through a series of incremental univariate tests, the next parameter to be added to the multivariate test, that is, the next fixed parameter that provides the largest increase in the multivariate *χ*^2^ statistic (see Bentler, 2006 for a review). To evaluate the model, the Satorra-Bentler parameter values were used, corrected by a robust covariance matrix, since these have been proven to provide the best adjustment indices (Bentler, 2006). In addition to the Satorra-Bentler Chi-square and its probability value (*p*), other fit indices used were the CFI (Comparative Fix Index), NFI (Normality Fix Index), NNFI (Non-Normality Fix Index) (≥0.90 suitable, ≥0.95 optimum), and RMSEA (Root Mean Square Error of Approximation) (≤0.08 adequate, ≤0.05 optimal) (Hu and Bentler, 1999).

## Results

### Attrition Analyses

At wave 1, Little’s MCAR test showed a non-significant chi-squared [*χ*^2^ = 233 (1), *p =* 0.63]. Regarding wave 2, similar results were obtained [*χ*^2^ = 859 (3), *p =* 0.83]. These results confirm completely at random nature of the missing data and it was therefore decided not to exclude them from the analyses.

### Psychological Well-Being During Adolescence: A Longitudinal Analysis

The first aim of this work was to analyze the level of psychological well-being during adolescence and its stability over time. As regards the overall scores, the correlations of paired samples reflected a positive association between both waves for all the dimensions, with moderate values ranging from 0.39 (positive interpersonal relationships) to 0.57 (self-acceptance) (Table 1). In all dimensions, the scores obtained represented medium-high levels of psychological well-being, given that they were greater than four points on a scale of 1–6. In both wave 1 and 2, the highest values corresponded to life development, and the lowest to autonomy (Table 2); the only significant differences were found in positive interpersonal relationships, which increased in time 2 [*t* (670) = −2.26, *p* = 0.024]. As regards the participants’ gender, the results showed the same tendency. The correlations found between both times showed a moderate, positive relationship between all the dimensions, with values ranging from 0.38 (positive interpersonal relationships in girls) to 0.60 (self-acceptance in girls) (Table 1). Both boys and girls showed higher levels of positive interpersonal relationships, autonomy and life development in wave 2, as well as lower levels of self-acceptance. Again, the results indicated medium-high values of well-being in all dimensions; in both wave 1 and 2, boys and girls had the lowest scores in autonomy, and the highest scores in self-acceptance (boys) and in life development (girls) (Table 2). The *t*-test for related samples showed that there were no significant differences between measurements in the first and second waves in boys, and so the scores were stable over time. In girls, significant differences were only found with respect to positive interpersonal relationships [*t* (344) = −1.99, *p* = 0.048].

**Table 1:** Psychological well-being correlations for paired samples.

	Self-acceptance		Positive interpersonal relationships		Autonomy		Life development	
Wave 1-Wave 2	*r*	sig	*r*	sig	*r*	sig	*r*	sig
Overall	0.57	0.000	0.39	0.000	0.41	0.000	0.40	0.000
Boys	0.52	0.000	0.41	0.000	0.31	0.000	0.39	0.000
Girls	0.60	0.000	0.38	0.000	0.51	0.000	0.42	0.000

**Table 2:** Means (SD) of psychological well-being.

	Self-acceptance	Positive interpersonal relationships	Autonomy	Life development
**Wave 1**				
Overall (*n* = 747)	4.77 (0.89)	4.69 (0.96)	4.03 (1.07)	4.87 (0.95)
Boys (*n* = 367)	4.90 (0.83)	4.67 (0.93)	4.03 (1.07)	4.76 (0.99)
Girls (*n* = 375)	4.64 (0.94)	4.71 (0.99)	4.03 (1.08)	4.97 (0.89)
**Wave 2**				
Overall (*n* = 747)	4.73 (0.99)	4.79 (1.01)*	4.08 (1.09)	4.88 (0.96)
Boys (*n* = 367)	4.85 (0.95)	4.74 (1.01)	4.10 (1.07)	4.84 (0.99)
Girls (*n* = 375)	4.61 (1.03)	4.83 (1.00)*	4.07 (1.12)	4.92 (0.92)

* *p < 0.05; missing data ranged from 4.82% (n = 36) to 5.62% (n = 42) across all variables*.

### The Association Between Romantic Relationships and Psychological Well-Being in Adolescence

The secondary aim of this work was to analyze the association between romantic relationships and the psychological well-being of adolescents. To do this, the data corresponding to wave 1 were used. Romantic relationship status was recoded and identified by the labels *current romantic relationship, past romantic relationship*, and *never romantic relationship.* The results showed that 27% of the participants had a current romantic relationship, 36.9% had a relationship in the past, but did not have it in the present, and 30.9% had never had a romantic relationship (missing cases = 5.1%). Descriptive statistics of psychological well-being according to romantic status can be observed in Table 3. The results of Chi-Square test showed no significant differences between the means of psychological well-being dimensions in relation to participants’ romantic relationship status.

**Table 3:** Means (SD) of psychological well-being by romantic relationship status.

	Self-acceptance	Positive interpersonal relationships	Autonomy	Life development
Current romantic relationship	4.79 (0.89)	4.70 (0.96)	3.92 (1.12)	4.90 (1.09)
Past romantic relationship	4.72 (0.94)	4.77 (0.94)	3.96 (1.14)	4.93 (0.85)
Never romantic relationship	4.82 (0.67)	4.60 (0.98)	4.16 (0.94)	4.83 (0.83)

*Missing data = 38 (5.1%)*.

Regarding to SEM analyses, the results obtained enabled us to establish a model in which romantic relationship status was found to be a reliable predictor of adolescent psychological well-being. Relationships between psychological well-being dimensions were established starting from a saturated model, in which correlation indices between all the dimensions were examined. Thereafter, introducing the predictor variable (romantic relationship status), several models were tested. Finally, we obtained a model in which direct associations between life development and self-acceptance, self-acceptance and positive interpersonal relationships, and positive interpersonal relationships and autonomy were the only ones that led to an adjusted model. Additionally, the Lagrange Multiplier Test (LM Test) was performed to explore the restrictions which could improve the model. Following the results provided for the stepwise procedure of this test and focusing on univariate increment statistics, that is, *χ*^2^ values that stood apart from the rest and probabilities <0.05, eight covariances were finally established between the measurement errors of the items 11, 12, 13, 14, 15, 16, 17, 18, and 22.

**Figure 1:**
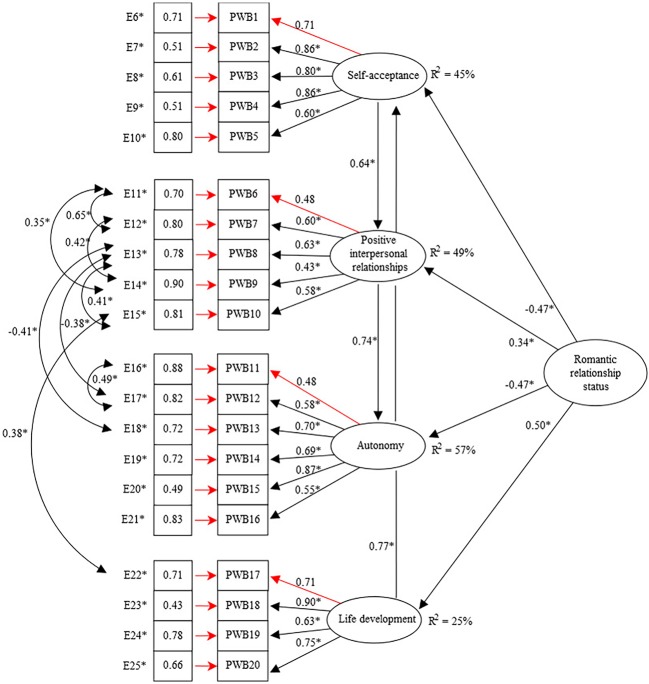
Romantic relationships and psychological well-being during adolescence Structural Equation Model (SEM) ^*^ *p* < 0.05.

A Mardia coefficient value of 121.9652 indicated that the data did not comply with the assumption of multivariate normality. The final model showed good adjustment indices, *χ*^2^ S-B = 757.0835; df = 174; *p* < 0.001; NFI = 0.941; NNFI = 0.951; CFI = 0.959; RMSEA = 0.059 [90% CI (0.053, 0.064)] (Figure 1). The results showed that there was a direct relationship between romantic relationship status and the four measurements of well-being assessed. A positive association with positive interpersonal relationships (*β* = 0.34; *p* < 0.05) and life development (*β* = 0.50; *p* < 0.05) was found and a negative one with self-acceptance (*β* = −0.47; *p* < 0.05) and autonomy (*β* = −0.47; *p* < 0.05). Likewise, it was found that life development was directly and positively related to self-acceptance (*β* = 0.77; *p* < 0.05), self-acceptance to positive interpersonal relationships (*β* = 0.64; *p* < 0.05), and positive interpersonal relationships to autonomy (*β* = 0.74; *p* < 0.05). These data showed that the dimensions where romantic relationship status had a greater impact were self-acceptance, autonomy, and life development, while its influence on positive interpersonal relationships was significant, but weaker. According to these results, adolescents who had a current or past romantic relationship were more likely to experience lower levels of autonomy and self-acceptance, but higher levels of life development and positive interpersonal relationships, compared to those who had never had a romantic relationship. In addition, higher levels of life development were associated with greater self-acceptance, higher levels of self-acceptance with better interpersonal relationships, and better interpersonal relationships with greater autonomy. As for the magnitude of the relationships between the variables, the explained variance (*R*^2^) for each of these relationships was from 25 to 57% (Figure 1).

## Discussion

Despite the evidence that the majority of adolescents overcome the challenges of this vital period without experiencing major social, emotional or behavioral difficulties (Steinberg and Morris, 2001), there are significantly fewer studies on adolescent well-being than those performed in the adult population (Huebner et al., 2009). Likewise, longitudinal studies regarding adolescent well-being adopt mainly a hedonic perspective, analyzing subjective well-being mostly in terms of satisfaction with life or affect balance (e.g., Inchley et al., 2016; Meade and Dowswell, 2016; Lerkkanen et al., 2018; Patalay and Fitzsimons, 2018), while only a few cross-sectional studies have been found adopting a eudaimonic approach (e.g., Loera-Malvaez et al., 2008; Ruini et al., 2009; Fernandes et al., 2010, 2011; Vescovelli et al., 2014; Opree et al., 2018). For these reasons, the first objective of this work was to analyze the level of psychological well-being during adolescence and its stability over time.

### Psychological Well-Being During Adolescence and Its Stability Over Time

The results obtained showed medium-high general levels in the four categories measured: *self-acceptance, positive interpersonal relationships, autonomy, and life development* (Viejo et al., 2018). These values remained stable, with the exception of positive interpersonal relationships, whose scores increased in wave 2. The adolescents who took part in the study maintained a positive perception of themselves over time, considered themselves capable of regulating their own behavior and felt that they were developing their potential. As time progressed, they considered themselves more capable of establishing close, trusting and meaningful relationships with other people, characterized by the expression of empathy, affection, and intimacy. The stability we found in psychological well-being did not allow us to support Hypothesis 1, according to which participants’ scores of well-being would decrease in time 2. These results may be related to studies claiming that eudaimonic activities produce longer-lasting effects on well-being, and that eudaimonic well-being is more stable and durable than hedonic well-being (Steger et al., 2008; Huta and Ryan, 2010). Nonetheless, this should be interpreted with caution, as no comparisons have been made in this study between the two types of well-being. On the other hand, results obtained go against the results of previous research in the adult population, which indicates that the levels of personal growth (termed *life development* in this study) decrease during this stage (Clarke et al., 2000; Springer et al., 2011). This is surprising, because although no significant differences were found between the two waves, the highest levels of psychological well-being found in this study corresponded to this dimension (life development) – with the exception of the boys in wave 1, who scored more high in self-acceptance, in both wave 1 and 2. Longitudinal studies with adolescent samples also provide contradictory results to those found in this research. The HBSC study (Inchley et al., 2016) and Patalay and Fitzsimons (2018) reported poor stability for well-being, which tended to decrease over time, while other authors found improvements or stable scores for well-being as time progressed (Meade and Dowswell, 2016; Lerkkanen et al., 2018). These results, however, must be taken with caution, since they focus mainly on hedonic variables (e.g., satisfaction with life). Despite the fact that we have not found longitudinal research which measures eudaimonic well-being in the same way, some cross-sectional studies have used Ryff’s psychological well-being scales (2014). Again, the results obtained are inconsistent compared to previous findings. Portuguese adolescents, for instance, showed both lower (Fernandes et al., 2010, study 1) and similar (Fernandes et al., 2010, study 2; Fernandes et al., 2011) levels of psychological well-being, while Dutch adolescents (Opree et al., 2018) and Italians (Ruini et al., 2009; Vescovelli et al., 2014) produced lower scores. This could be related to the data obtained by Ottová-Jordan et al. (2015), who found differences between the different countries analyzed.

As regards the participants’ gender, both boys and girls showed medium-high levels of well-being in all the categories evaluated; however, the boys felt more self-satisfaction than the girls, while the latter showed a greater feeling of improvement and development of their self-potential, in indicators which remained stable. As time progressed, the girls reported higher levels of positive interpersonal relationships. This result partially supports Hypothesis 1 formulations that stated the absence of gender differences at time 1 but not in the follow-up. Previous cross-sectional studies using similar measurements did not find any significant differences according to the participants’ gender (Vescovelli et al., 2014), while longitudinal studies which analyzed well-being in terms of satisfaction with different areas of life (e.g., friends, family, appearance, school, school work, and life as a whole) found contradictory results, indicating that girls showed a greater tendency to lose well-being compared to boys (Inchley et al., 2016; Meade and Dowswell, 2016; Booker et al., 2018; Patalay and Fitzsimons, 2018). Research in adult samples showed similar results to those found in this work, with women scoring higher than men in positive relationships with others and in life development (originally termed “personal growth” by Ryff) (Keyes et al., 2002; Lindfors et al., 2006; Ryff and Singer, 2008).

All together, these results suggest, in line with other studies, that well-being during adolescence has its own idiosyncrasy (Casas, 2010), which should be studied from approaches focused on its evolution and specific characteristics. In this regard, it is necessary to highlight as a key aspect the type of measurement used to evaluate it. The contradictory results produced by research could point to the need to develop a theoretical framework in which the empirical data would enable us to understand adolescent well-being as a whole. In addition, the differences shown in the studies in different countries may indicate the need to take cultural factors into consideration.

### Psychological Well-Being and Romantic Relationships During Adolescence

According to the specialized literature, well-being during adolescence is strongly influenced by the contexts in which adolescents develop (Geldhof et al., 2013; Inchley et al., 2016), and there is currently a sufficient body of scientific evidence on the impact of peers, parents, the neighborhood or the school on positive development (Benson and Faas, 2014). However, romantic relationships, despite being a particularly relevant context for adolescent well-being (Collins and Van Dulmen, 2006), have received little empirical attention (Steinberg and Morris, 2001; Collins, 2003). The secondary aim of this work therefore focused on analyzing the impact of these relationships on adolescent psychological well-being.

The results obtained show that romantic relationship status is a strong predictor of the four dimensions of psychological well-being measured. Participants with current or past romantic relationships showed better levels of positive interpersonal relationships and life development, while those who had never had a romantic relationship had higher levels of self-acceptance and autonomy. These results partially support Hypothesis 2, according to which romantic relationships will significantly predict adolescent psychological well-being, showing a positive association with self-acceptance, positive interpersonal relationships and life development dimensions, and negative with autonomy. The challenging nature of romantic relationships could account for the negative association found between romantic relationships and self-acceptance and autonomy dimensions. Following the postulates of the *stress and coping model* (Larson et al., 1999), adolescents may not have sufficient resources or coping strategies to deal with the demands of these new contexts, and since they do not feel capable of responding effectively, this could increase the risk of negative consequences (Davila, 2008), such as a more negative image of themselves and their own abilities. Some authors have suggested that novice learners can learn to develop awareness of their own metacognitive deficit (Miller and Geraci, 2011), in which the effect of the learning curve is especially important (i.e., the improvement in task response effectiveness as it is repeated). It is obvious that a novice clearly takes time to adjust to a new task and requires a settling-in time until the adjusted response is obtained (Mayer, 2011). As adolescents become involved in these new social exchanges and become aware of the skills, abilities, and competencies required, they may feel insecure and underestimate their ability to cope with them, which may lead to an *achievement plateau* which sows an element of doubt in their minds about their own competence. It is reasonable, therefore, to deduce that romantic relationships have differential effects on the psychological well-being of adolescents, since the learning involved is usually self-taught and the immediate context is rarely involved. This could be linked to the results of previous research, which has found associations between romantic relationships and physical and mental problems (Davila et al., 2016). Other studies have associated romantic relationships to another type of negative consequences, such as violence within the couple, substance use, poor school performance or internalizing and externalizing behaviors (e.g., Zimmer-Gembeck et al., 2001; Ackard and Neumark-Sztainer, 2002; Callahan et al., 2003; Cui et al., 2012; Viejo et al., 2015). However, in the specific filed of well-being, it is important to emphasize that no studies has been found analyzing the influence of romantic relationships in adolescent well-being following a eudaimonic approach. Studies focused on psychological well-being frequently understand it as the absence of depression and anxiety symptoms (e.g., Callahan et al., 2003; Bauermeister et al., 2010; Baams et al., 2014; Miller, 2014) and associate adolescent romantic relationships mainly with high levels of them (Callahan et al., 2003; Miller, 2014). These results are contradictory to the findings of this work, probably due to the measures used and the different approaches to the study of well-being. The large disparity between the conceptualization of psychological well-being as a multidimensional construct focused on what means to be healthy, well and fully functioning (Ryff and Singer, 2008), and the opposite one, which understand it mostly in terms of absence of illness or mental disorders, makes the task of drawing conclusions particularly difficult.

Besides this, the data obtained could point to a double reading of the impact of romantic relationships on adolescent well-being. Thus, the higher rates of life development and positive interpersonal relationships in the participants with romantic relationships could lead us to the idea that their challenging nature can equally lead to positive outcomes. As well as to being a developmental challenge, they can also act as a stimulating context in which to acquire skills for coping and managing interpersonal relationships. Therefore, as adolescents become involved in these types of relationships, they may eventually feel that they are improving and developing their potential and increasing their capacity to maintain close, trusting relationships based on empathy. These findings are consistent with those from other research, which has shown that romantic relationships provide a context of great intimacy, support, and importance for their protagonists (Demir, 2010; Shulman et al., 2011) and are a source of emotional connection and social integration (Meirer and Allen, 2008). It is also important to stress the links found between autonomy, romantic relationships, and positive interpersonal relationships. While romantic relationships were negatively related to autonomy, positive interpersonal relationships had a positive link; in other words, maintaining satisfactory relationships with peers had positive effects on the adolescents’ ability to feel independent and follow their own convictions, while romantic relationships had the opposite effect. These results could point to the distinctive nature of romantic relationships in comparison to other types of relationships, such as friendship (Collins, 2003; Collins et al., 2009). They seem to have a specific influence on adolescent well-being, fact that suggests the need to understand them as specific phenomena with their own characteristics.

Altogether, this calls for a need to take actions to promote healthy relationships; in other words, a need for training young people to manage and acquire the skills they need to maintain satisfactory relationships (Davila et al., 2017). Maintaining romantic relationships requires specific abilities which differ from those learned and used in other types of interpersonal relationships (Shulman et al., 2011). From this point of view adolescence, a stage in which erotic-sentimental interest begins and the first romantic relationships take place can be seen as the best period to start taking this kind of steps (Shulman et al., 2011).

### Limitations and Future Directions

One limitation of this work is that, despite of being a longitudinal study, the follow-up period was relatively brief. On the other hand, the consideration of participants’ romantic status through a cross-sectional design, calls for further longitudinal studies taking into account the impact of specific characteristics of romantic experience on adolescent well-being, such as the duration of the relationships, the content of the interactions or the romantic status changes. Therefore, cross-lagged path analyses would be very useful to compare the prospective relationships between romantic relationships and well-being. Concerning the possible gender and age moderation in the association between romantic relationships and adolescent well-being, in future studies, it would be appropriate to carry out multigroup analyses that help to clarify this issue. In addition, it is also suitable to control other variables, such as relationships with parents and peers. Besides that, the use of self-reports on aspects related to well-being could be affected by a *positivist bias*, that is, the tendency to overestimate the real values of well-being (Cummins, 1998). Future studies in populations outside Spain could also further our understanding of adolescent well-being and help us to draw more general conclusions about how it works and about its characteristics. Finally, without denying the significance of studying the implications of romantic relationships for adolescents’ mental illness, the confusing scenario found with respect to psychological well-being conceptualization suggests the need to continue making empirical efforts to consolidate a shared language in well-being arena, as well as to strengthen the notion that mental health is not the mere albescence of mental illness (Seligman, 2008).

## Conclusions

The reduced amount of studies in comparison with adulthood, in both romantic relationships and eudaimonic well-being, suggests that further research is required to help shed light on the functioning, characteristics, and associations of these two phenomena during adolescence. This study provides empirical support for the view of adolescence as a period with good levels of well-being, as well as providing evidence of its stability. Furthermore, it reinforces the consideration of romantic relationships as essential contexts for adolescent well-being (Collins and Van Dulmen, 2006; Connolly and McIsaac, 2009). In this sense, it is important to emphasize that the practical implications of research on romantic relationships in adolescence and well-being must take into account the fundamental importance of the contexts in which adolescents develop. A greater involvement of parents, teachers, clinician, and adults in general would be essential to achieve optimal results. Romantic relationships have an effect on the well-being of adolescents, and the issue of how we, as members of the adult world, can help ensure that this effect is positive should not be avoided (Collins, 2003). Most parents, teachers, and clinicians will probably have to deal on a daily basis with issues related to romantic relationships of their children, students or patients and may feel overwhelmed or constrained in their capacity to provide guidance (Florsheim, 2003). In this regard, it is crucial that adults acquire knowledge about how to help adolescents to maintain healthy romantic relationships (Florsheim, 2003), given their significant influence on adolescent well-being.

Particularly in relation to well-being, a change of mindset about adolescence is also considered necessary. It is essential that the potentialities of adolescents, their positive qualities, their capacity to achieve a successful adjustment, and feel high levels of well-being be taken into account in the different areas with the potential to promote a positive development during these years: family, school, community, clinical practice, and scientific research. Only under this positive vision of adolescence, it will be possible to design and develop research and intervention programs encouraging knowledge and involvement of adults in adolescent romantic context, promoting well-being of boys and girls and teaching them about how to successfully manage their relationships, thus creating social and empirical means to promote a positive adjustment in these years leading to adulthood.

## Data Availability

The datasets generated for this study are available on request to the corresponding author.

## Ethics Statement

This study was carried out in accordance with the recommendations of the Declaration of Helsinki and the Spanish Society of Psychology. The protocol was approved by the Comité de Bioética y Bioseguridad de la Universidad de Córdoba (Bioethics and Biosafety Committee of the University of Cordoba). The study was approved by the school boards, and the consent obtained from the parents of the participants was both written and informed.

## Author Contributions

All authors listed have made a substantial, direct and intellectual contribution to the work, and approved it for publication.

### Conflict of Interest Statement

The authors declare that the research was conducted in the absence of any commercial or financial relationships that could be construed as a potential conflict of interest.
